# Factors affecting actualisation of the WHO breastfeeding recommendations in urban poor settings in Kenya

**DOI:** 10.1111/mcn.12161

**Published:** 2014-12-17

**Authors:** Elizabeth W. Kimani‐Murage, Frederick Wekesah, Milka Wanjohi, Catherine Kyobutungi, Alex C. Ezeh, Rachel N. Musoke, Shane A. Norris, Nyovani J. Madise, Paula Griffiths

**Affiliations:** ^1^ African Population and Health Research Center (APHRC) Nairobi Kenya; ^2^ Department of Paediatrics University of Nairobi Nairobi Kenya; ^3^ MRC/Wits Developmental Pathways for Health Research Unit Faculty of Health Sciences University of the Witwatersrand Johannesburg South Africa; ^4^ Centre for Global Health, Population, Poverty, and Policy University of Southampton Southampton UK; ^5^ Centre for Global Health and Human Development Loughborough University Loughborough UK

**Keywords:** exclusive breastfeeding, infant feeding behaviour, breastfeeding duration, breastfeeding knowledge, child nutrition, urban slums

## Abstract

Poor breastfeeding practices are widely documented in Kenya, where only a third of children are exclusively breastfed for 6 months and only 2% in urban poor settings. This study aimed to better understand the factors that contribute to poor breastfeeding practices in two urban slums in Nairobi, Kenya. In‐depth interviews (IDIs), focus group discussions (FGDs) and key informant interviews (KIIs) were conducted with women of childbearing age, community health workers, village elders and community leaders and other knowledgeable people in the community. A total of 19 IDIs, 10 FGDs and 11 KIIs were conducted, and were recorded and transcribed verbatim. Data were coded in NVIVO and analysed thematically. We found that there was general awareness regarding optimal breastfeeding practices, but the knowledge was not translated into practice, leading to suboptimal breastfeeding practices. A number of social and structural barriers to optimal breastfeeding were identified: (1) poverty, livelihood and living arrangements; (2) early and single motherhood; (3) poor social and professional support; (4) poor knowledge, myths and misconceptions; (5) HIV; and (6) unintended pregnancies. The most salient of the factors emerged as livelihoods, whereby women have to resume work shortly after delivery and work for long hours, leaving them unable to breastfeed optimally. Women in urban poor settings face an extremely complex situation with regard to breastfeeding due to multiple challenges and risk behaviours often dictated to them by their circumstances. Macro‐level policies and interventions that consider the ecological setting are needed.

## Introduction

Undernutrition is a significant public health concern, and reducing it is therefore one of the Millennium Development targets for 2015 and also a World Health Assembly target for 2025. Undernutrition is associated with adverse short‐term and long‐term effects on child health, development and survival. It is associated with increased morbidity and mortality, hence increased burden of disease, mental and motor development, and increased risk of obesity and metabolic diseases later in the life course (Oddy *et al*. 2003; Grantham‐McGregor *et al*. 2007; Victora *et al*. 2008; Lanigan & Singhal 2009; World Health Organization 2009). High levels of undernutrition have been documented in low‐income countries despite many global strategies, declarations and policies aimed at combating it (World Health Organization 2003,2007). For example, in sub‐Saharan Africa (SSA), approximately 40% of all children under 5 years (56 million) are estimated to be stunted (United Nations Children's Fund *et al*. 2012). Wasting, including severe wasting with implications on child survival, is also prevalent in low‐income countries. For example, a study by Kerac *et al*. (2011) using data from Demographic and Health Surveys indicated that among 21 developing countries, prevalence of wasting for children under 6 months may be as high as 34%. In Kenya, high levels of undernutrition have been documented. At the national level, 35% of children under 5 years are stunted, 16% are underweight, and 7% are wasted (Kenya National Bureau of Statistics & ICF Macro 2009). The problem of undernutrition is even worse in urban poor settings with stunting prevalence of over 40% (Olack *et al*. 2011; Abuya *et al*. 2012).

Causes of malnutrition are grouped into three main categories: immediate, underlying and basic (United Nations Children's Fund 1990; Engle *et al*. 1999). The immediate causes include inadequate dietary intake and health status of the child. The underlying causes include food insecurity, child care practices, health service delivery and environment. The basic causes include economic, political and ideological structures. In line with this model of care, there is a growing recognition of the importance of nutrition in the first 1000 days of life from conception with regard to child growth, health and survival (Isolauri *et al*. 2011). The global strategy on infant and young child nutrition (IYCN) highlights the notion that inadequate knowledge about proper foods and feeding practices is often a more important determinant of malnutrition than the availability of food (World Health Organization 2002,2003). It is estimated that interventions that promote optimal breastfeeding and complementary feeding could prevent about a fifth of under five deaths in countries with high child mortality rates (Gareth *et al*. 2003; Kramer & Kakuma^2004^). Poor care practices during the first 1000 days of life have been widely documented in the low‐ and middle‐income countries (LMICs). For example, about 40% of infants in LMICs are exclusively breastfed for the first 6 months (Lauer *et al*. 2004). In Kenya, a third of children are exclusively breastfed for the first 6 months while about 40% of children aged 6–23 months are fed according to IYCN guidelines (Pan American Health Organization 2003; World Health Organization 2005). In urban poor settings in Kenya, poor infant feeding practices have been identified. While close to 40% of the infants are not breastfed within 1 h following delivery, only 2% are exclusively breastfed for the first 6 months, and 15% stop breastfeeding by the end of 1 year (Kimani‐Murage *et al*. 2011). As a possible consequence of poor infant feeding practices among other potential causes such as poor water and environmental sanitation and access to health services (African Population and Health Research Center 2002a,b; Kimani‐Murage & Ngindu 2007), high levels of malnutrition have been documented among urban poor residents with stunting prevalence of over 40% (Abuya *et al*. 2012).

Various quantitative studies in developing countries have identified factors associated with suboptimal breastfeeding and complementary feeding practices. These include maternal characteristics such as age, marital status, occupation and education level; antenatal and maternity health care seeking; health education and socio‐economic status; and the child's characteristics including birthweight, method of delivery and birth order, but these findings are conflicting with regard to the consistency of the associations and the magnitude of the effects (Morisky *et al*. 2002; Pascale *et al*. 2007; Akter & Rahman 2010; Patel *et al*. 2010; Kimani‐Murage *et al*. 2011; Setegn *et al*. 2012). This suggests that the context is important when trying to isolate characteristics and practices that may be amenable to interventions. In urban poor settings in Kenya, a study by Kimani‐Murage *et al*. (2011) found that factors associated with suboptimal infant feeding practices include child level factors such as child's sex and perceived size at birth; maternal characteristics including marital status, ethnicity and education level; and other factors including whether pregnancy was desired or not, and place of delivery (Kimani‐Murage *et al*. 2011). Through qualitative studies, perceptions regarding the factors affecting the practices have also been explored. Structural and social‐cultural factors including food insecurity, lack of knowledge or competence, socio‐cultural myths and health status of the mother or the baby have been identified among other factors (Webb‐Girard *et al*. 2012; Matsuyama *et al*. 2013; Ijumba *et al*. 2014). However, there is limited evidence, particularly qualitative studies explaining the factors influencing breastfeeding and other infant feeding practices in urban slums because few studies have focused on urban slums. This is despite the fact that urban slums are becoming increasingly important as majority (approximately 62%) of urban residents in SSA live in such slums (United Nations Human Settlements Programme 2008), with very unique circumstances and complexities that may have implications on infant feeding and care practices and the nature of the factors that influence these practices. This study therefore investigates the factors that contribute to poor breastfeeding practices in two urban slums in Nairobi, Kenya, using qualitative methods.

### Key messages

- General awareness regarding optimal breastfeeding practices exists in urban poor settings. However, this knowledge is not translated into practice, leading to sub‐optimal breastfeeding practices.
- Women in urban poor settings face an extremely complex situation with regards to breastfeeding due to multiple challenges and risk behaviors often dictated to them by their circumstances and context.
- While individual level interventions to change behaviour may be important, macro‐level policies and interventions that take into consideration the ecological setting are particularly needed in order to improve breastfeeding behaviours among urban poor women.

## Methods

### Study setting and population

The study was conducted in two slums of Nairobi, Kenya, namely Korogocho and Viwandani. These were purposively selected because of the presence of a routine urban Demographic Surveillance System, which is managed by the African Population and Health Research Center (APHRC) (Emina *et al*. 2011). The two slums are located about 7 km from each other. They occupy a total area of slightly less than 1 km^2^ and are densely populated with 63 318 and 52 583 inhabitants per square km, respectively. Because they are not recognised as legal settlements, provision of basic services is a complex issue and is not considered a government obligation. The slums have poor housing, no basic infrastructure such as potable water and waste disposal, and are characterised by high levels of violence and insecurity, unemployment and poor health indicators (African Population and Health Research Center 2002a; Fotso *et al*. 2009; Emina *et al*. 2011; Kimani‐Murage *et al*. 2011; Fotso *et al*. 2012). Viwandani, being located in the industrial area, attracts migrant workers especially men with relatively higher levels of education. The area has a higher proportion of households with only one person compared with Korogocho. Korogocho, on the other hand, has a more stable population and greater co‐residence of spouses but higher unemployment levels (Emina *et al*. 2011). There are several ethnic groups in the areas including the Kikuyu, Kamba, Luo, Luhya and Somalis, with Swahili spoken by all ethnic groups. While each ethnic group harbours specific cultural norms, beliefs and practices with regard to maternal, infant and young child feeding, these have been watered down by inter‐cultural ethnic relationships and ‘urban life’.

### Data collection

In April 2012, a total of 19 in‐depth interviews (IDIs), 10 focus group discussions (FGDs) and 11 key informant interviews (KIIs) were conducted in Korogocho and Viwandani. Participants were recruited through purposive sampling depending on category of respondents, taking into account different ethnicity, religious affiliation and village of residence. Community mobilisers, who are well known in the community, assisted in recruiting the participants. In total, there were 110 participants including 20 men and 90 women from the following categories: (1) women of reproductive age who were either pregnant, breastfeeding or had children under 5; (2) community leaders including village elders, women leaders, youth leaders and religious leaders; (3) health care professionals; (4) community health workers; and (5) traditional birth attendants (TBAs). Other characteristics of the respondents are given in Tables 1 and 2.

**Table 1:** Interviews by type and category

Interviews/study site	Korogocho	Viwandani	Total
By type of interview (*n* = interviews)			
In‐depth interviews	11	8	19
Focus group discussions	6	4	10
Key informant interviews	6	5	11
By category (*n* = interviews)			
Focus group discussions			
Village elders	1	1	2
Young mothers (below 25 years old)	2	1	3
Older mothers (25+ years old)	2	1	3
CHW	1	1	2
In‐depth interviews			
Pregnant mothers	2	1	3
Breastfeeding mothers	5	4	9
Mothers of children under 5 years old (not breastfeeding)	2	1	3
HIV‐positive mothers	2	2	4
Key informant interviews			
Health care workers	1	1	2
Religious leaders	1	1	2
Traditional birth attendants	1	1	2
Women leaders	2	1	3
Youth leaders	1	1	2

CHW, community health worker.

**Table 2:** Socio‐demographic characteristics of participants

Socio‐demographic characteristics (*n* = individuals)	Men	Women	Total
Age			
Mean age	41.8	29.3	31.58
<25 years	1	39	40
≥25 years	19	51	70
Religion			
Christian	19	74	93
Muslim	1	14	15
Missing	0	2	2
Ethnic background			
Kikuyu	2	25	27
Kamba	5	17	22
Luo	4	23	27
Luhya	4	9	13
Somali	0	4	4
Other	5	12	17
Education status			
None	0	7	7
Pre‐primary (early child development)	4	11	15
Primary	4	50	54
Secondary	7	18	25
Post‐secondary/college	5	4	9
Occupation			
Casual worker	0	11	11
CHW	1	9	10
Health worker (nurse, clinical officer)	1	2	3
Social worker/religious leader	3	0	3
Self‐employed/business including artisans	11	27	38
Community leader	4	0	4
Not working (including housewife, student)	0	37	37
Missing	0	4	4
Marital status			
Married	18	50	68
Widowed	0	4	4
Not married/single/separated	2	34	36
Missing	0	2	2
Slum			
Korogocho	8	59	67
Viwandani	12	31	43

CHW, community health worker.

The study used open‐ended questions, with focus on getting deeper understanding regarding factors that influence IYCN focusing on the World Health Organization (WHO) recommendations for breastfeeding and other IYCN. Pictures of children depicting nutritional status and of foods were used to stimulate responses. Questions included perceptions on the nutritional status of the majority of infants living in the local community; knowledge, attitudes and practices with regard to maternal, infant and young child nutrition (MIYCN) including initiation of breastfeeding, use of colostrum, exclusive breastfeeding, duration of breastfeeding and complementary feeding. Additionally, questions focused on the contextual and socio‐cultural norms that influence the practices. Socio‐demographic characteristics of the participants were recorded ahead of the interviews or focus group discussions.

Interviews were conducted by 10 experienced field interviewers (seven female and three male) with undergraduate training in nutrition, public health, sociology or anthropology. Pilot interviews were conducted during the training sessions. Debriefing sessions were held to ensure consistency of meaning to questions. New issues and emerging themes, for example, regarding breastfeeding by HIV‐positive women, were added to the interview guide and explored in subsequent interviews. Some of the researchers accompanied the field team in pilot interviews and participated in the debrief sessions. There was always an interviewer/moderator and a note taker in each interview or FGD. Interviews were conducted in Swahili. All interviews were audiotaped and transcribed verbatim. Concurrent transcription and translation was done by two graduates with good experience in anthropology and transcription who had participated in the training of the interviewers and the pilot sessions.

### Ethical considerations

The study was granted ethical approval by the Kenya Medical Research Institute Ethical Review Committee. All the investigators have had research ethics training. Participation was voluntary, and informed consent was sought from all the respondents.

### Data analysis

Themes were developed from literature and from the narratives from the respondents. The researchers familiarised themselves with the data by listening to audio tapes and reading the transcripts. Transcribed Microsoft Word files were imported into NVIVO 10 software (QSR International Pty Ltd, Doncaster, Victoria, Australia), which helped identify primary and meta codes and major themes. Coding and interpretation was done by two members of the research team to ensure objectivity and to check for consistency in application of the coding process. Final checks for understanding and consistency of the application of the codes were undertaken with a third member of the research team. Analysis across all transcripts was done thematically (Lacey & Luff 2001). We used the conceptual framework of factors affecting breastfeeding practices developed by Hector *et al*. (2005) to guide data analysis and to summarise our study findings into a modified conceptual framework.

## Results

### Knowledge, perceptions and practices regarding breastfeeding

There was mostly good general knowledge regarding the optimal breastfeeding practices across all the different categories of respondents involved in the study. Respondents were aware of the benefits of immediate initiation of breastfeeding after birth and exclusive breastfeeding for 6 months. However, there were also some gaps in knowledge, myths and misconceptions regarding breastfeeding.

#### Immediate initiation of breastfeeding

There was general consensus that children should be given breast milk immediately after birth if there are no maternal complications such as Caesarean section birth and/or mother's illness. Positive views regarding colostrum were also expressed, but the positive views seemed to be more recent, resulting from education on the importance of colostrum and immediate initiation of breastfeeding:

For me, the moment I give birth and I am given my baby, I breastfeed the baby so as to get the yellow milk, It helps the baby's brain development. I'm speaking about my practice. (FGD, Older Mothers, Korogocho)

For me, I used to see it [colostrum] being expressed out before the baby is breastfed. But recently I see them breastfeeding the babies on it. I don't know because they are saying it's medicine. I can't know because I am a man. (FGD, Community Health Workers, Korogocho)

#### Exclusive breastfeeding

There was general consensus that children should be breastfed exclusively for the first 6 months among the majority of the respondents. Some of the reasons given were: ‘it is a child's right’, ‘that is what the doctors say’, ‘the mother's milk has everything; always ready and uncontaminated’, ‘children grow well and are healthier’, ‘it will even save one from the hospital bills’, ‘children become very clever’, ‘the mother gets back to shape quickly’ and ‘it helps in family planning’. ‘We hear from the doctor. The doctors tell us it is good to breastfeed. If you breastfeed the child becomes healthy. And if you breastfeed for six months without giving the baby anything else, the baby's health will be good’ (IDI, Breastfeeding Mother, Korogocho).

However, the respondents cited unique constraints to exclusive breastfeeding that make it impractical particularly related to livelihoods. Therefore, practice is not in sync with the expressed views regarding exclusive breastfeeding because of work‐related issues, misperceptions, and other social and structural barriers described below under factors.

The one which is recommended is six months, but here, that six months does not happen … After one month the mother takes the baby to a child care center and she goes to work … Will she stay in the house and she has nothing to eat? (FGD, Village Elders, Viwandani)

For some, milk alone cannot be enough … You will have to boil for them water because they cannot get satisfied with that milk. If you give them breast milk, they continue to cry because they are not satisfied, so you will have to boil water for them. (IDI, Breastfeeding Mother, Korogocho)

#### Continued breastfeeding

With regard to the duration of breastfeeding, there were mixed views as to how long children should be breastfed. Duration of breastfeeding was said to be highly variable with some not breastfeeding at all, others breastfeeding for a few months, and others for a year, 2 years and beyond 2 years. The shortest duration was perceived to be among young mothers, working mothers and women participating in commercial sex work.

### Factors affecting breastfeeding in urban slum settings

**Figure 1:**
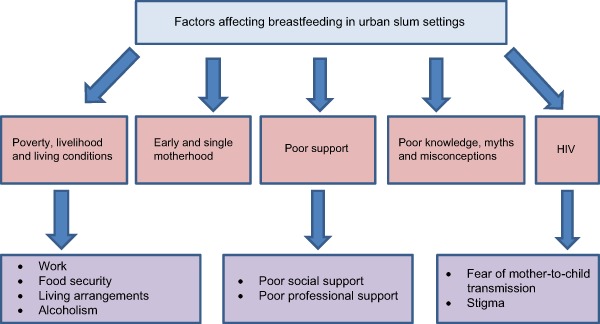
Themes and sub‐themes on factors affecting breastfeeding in urban slum settings.

**Figure 2:**
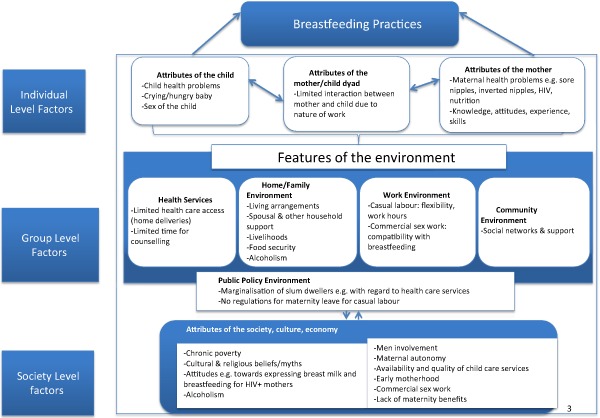
Conceptual framework of factors affecting breastfeeding practices in urban slums (adapted from Hector *et al*. 2005).

Various social and structural barriers were said to influence breastfeeding practices, hence making it impractical to actualise WHO recommendations for breastfeeding. These factors are outlined in a schematic diagram of themes and sub‐themes (Fig. 1) and summarised in a draft conceptual framework (Fig. 2), adapted from Hector *et al*.'s (2005) conceptual framework of factors affecting breastfeeding practices. The draft conceptual framework proposes three levels of factors that influence breastfeeding practices in the urban slum settings: individual level factors, relating directly to the mother, child and the ‘mother‐child dyad’; group level factors, constituting the attributes of the environments where the mother and the child live which enable the mother to breastfeed; and the society level factors that influence the acceptability and expectations regarding breastfeeding and provide the context for breastfeeding.

Below we describe in more detail the key factors that influence breastfeeding practices in the study community, organised thematically to include: (1) poverty, livelihood and living conditions; (2) early and single motherhood; (3) poor social and professional support; (4) poor knowledge, myths and misconceptions; (5) HIV; and (6) unintended pregnancies.

### Poverty, livelihood and living conditions

#### Work

Mothers were reported to resume work shortly after giving birth because ‘the baby won't eat the name “good care” ’ (FGD, Young Mothers, Korogocho), and the mother and her family must also survive. Women work long hours in non‐conducive environments for carrying babies to work or breastfeeding. The child is therefore left behind either under the care of siblings, other relatives, neighbours or at a (sub‐standard) day care center.

At times it's the challenge of work; you are supposed to breastfeed, yet you are supposed to go to work. The mother gets problems, and the way life is hard nowadays, you are forced to go fend for yourself whether you have a baby or not. So you have to leave the baby. (FGD, Older Mothers, Viwandani)

Nowadays, mothers take their children to baby care centers even when they are one month. This child is started on foods when the stomach is not yet strong for food and so the intestines will expand. The baby care attendant cannot take good care of the child since she has about 20 of them to look after. Baby care centers are the reason why children are malnourished especially in our village since the centers have many children; a mother is better off looking for someone to take care of the child in the house. (KII, TBA, Viwandani)

Expressing breast milk to feed the baby was not a common practice, and in some instances it was considered culturally unacceptable. However, a few mothers had attempted to express, but either did not yield much or it was too painful. Some expressed milk to throw away if the breasts were too full, while others indicated that mothers should express and give the milk to the baby if the breasts had sores. This was particularly considered important for HIV‐positive women to prevent infecting the baby.

I have never heard of mothers (in this community) expressing breast milk. But we tell them as they continue breastfeeding, to alternate the breasts which baby breastfeeds and to express milk when the baby is asleep. They refuse and say culturally it's not right to express breast milk. They say the ancestors will be angry (if they express) then milk will reduce. (KII, Health Professional, Viwandani)

In my situation, if the breast has some sores, I can use water to express the milk in a cup and give it to the baby while it is still warm. (IDI, HIV‐Positive Mother, Viwandani)

While some mothers may be open to expressing breast milk so as to leave it behind when they go to work, there is concern regarding preservation of the milk as most households do not own refrigerators in these slum settings, and also concern on how to warm it.

It [expressing breast milk] is good if you have enough milk because it is hard to always be with the baby. We should also be taught on ways of preserving because it usually changes the smell and consistency after some time, and it is also – destroyed by direct heat. And you will find that here in this community, many parents do casual jobs and are never with their children. (IDI, HIV‐Positive Mother, Korogocho)

The only viable option for working mothers seems to be leaving breast milk substitutes, especially cow milk and porridge, for their babies regardless of their age because formula milk was considered too expensive: ‘The money! Will you pay rent or buy milk? … The price is too high!’ (FGD, Young Mothers, Korogocho). Children of working mothers were said to forget how to breastfeed or refuse to breastfeed as they stay for long hours without breastfeeding, so ‘they are used to the grandmother and are not interested in the mother’ (FGD, CHWs, Korogocho).

Some respondents indicated a need for paid maternity leave, in line with the constitution, to allow women to breastfeed optimally, while others recommended material support such as cash transfer or food donation for mothers to enable them time to breastfeed.

Commercial sex work was mentioned as an important source of livelihood in these communities: ‘You find there is a lot of prostitution’ (KII, Religious Leader, Korogocho). This work was not considered conducive for breastfeeding as women feared that the baby would get used to breastfeeding, and the business would be affected. There was also a fear of milk leaking onto the blouse, breasts would sag/flatten, or they would age faster, which would make them less marketable. Finally, commercial sex work was demanding with regard to time. Sometimes, infants were given sleeping pills or alcohol to make them sleep the whole night while mothers worked.

They don't even have time … when going for dates or as commercial sex workers, they leave the child with a friend who may not take good care of the child. Sometimes they give alcohol to the child for overnight escapades; this way the child is locked in the house, will sleep and will not even feel hungry till they return in the morning. (KII, Women leader, Korogocho)

#### Food insecurity

Exclusive breastfeeding for 6 months was said to happen only for those who had sufficient food at home. There was a general feeling that mothers do not eat enough food as there is limited food in the household, and therefore are not able to produce enough milk.

… And many if you ask them, they will ask you how they will breastfeed and they haven't eaten. And if you look at the breasts, they have flattened and are sagging, and they are girls. You can give them advice the way we've said, but if you go to their house they have nothing to eat. So even if you tell them to breastfeed, first of all that milk is not there because there is no food in the house. (FGD, Village Elders, Viwandani)

If you eat well, breast milk alone can be adequate but if you do not eat well it will not be adequate … You find that there are some mothers who go to hustle and you see that (food) will not be enough. (IDI, Young Mother, Korogocho)

#### Living arrangements

The small (10 × 10 feet) multipurpose one‐room houses, often accommodating all of the household members, do not offer a conducive environment for breastfeeding. The option of the mother sleeping with the baby on the same bed sometimes raises conflict with her spouse as the bed is also small. Mothers are often forced to stop breastfeeding prematurely.

The type of house, in this case the one room structures in this community, may make the mother to stop breastfeeding. The rooms are small and everything is cluttered within it, a bed next to another and they serve as the kitchen, bedroom, and sometimes the bathroom. If the baby cries at night to breastfeed, it disturbs everyone and the mother may decide to stop breastfeeding early. (FGD, Village Elders, Korogocho)

#### Alcoholism

Male alcoholics neglect the family and domestic violence is common causing a lot of stress to the breastfeeding mother. Women become the breadwinners and hence have no adequate time to breastfeed. Alcoholism in women makes them neglect their children, as ‘she (the mother) will come and sleep (after drinking), and when she's sleeping the child will be crying outside’ (KII, Women Leader, Viwandani).

Here in Korogocho, I can say it [alcoholism] is because of the prevailing problems. Even if you have a husband, he is jobless and many times when you go to these houses it is mostly the women who work. Most men don't work; they are just drunkards and do casual jobs … The mothers will have no choice but to stop breastfeeding so as to look for work in order to feed themselves. Even the stress, tiredness, you will find that when the mother reaches home she is not even able to breastfeed. Most of them know the importance but you will just hear someone tell you they are forced to stop breastfeeding. (KII, TBA, Korogocho)

### Early and single motherhood

Teenage motherhood, which is common in the areas, was considered a key factor in breastfeeding practices. The young and single mothers were said to be highly concerned about body image which influenced their breastfeeding practices.

There are many ‘dotcoms’ (young girls – born in the era of internet) in this community, sometimes we call them ‘face value’ … or ‘Facebook’ … The youths do not like to breastfeed, and so will take their children to baby care centers. They buy ready‐made rice to be given to the child, this is not nutritious food and so the child does not gain, in fact the child feeds like an adult. If you tell them to exclusively breastfeed up to six months they disagree as they claim the breasts will sag. (FGD, Community Health Workers, Viwandani)

They were also said to be often still in school or have busy lifestyles as they still have many friends, and are still looking out for fun. Some girls do not even initiate their babies on the breast, as introducing the baby to breast would mean the baby would get too used to the breast yet they need to go back to school or continue with their busy lives.

Here in Korogocho, there are many girls who give birth while still in school, so they start the baby on bottle feeding or porridge so that they can go back to school. I have seen that from my neighbor … Girls in this area don't have a particular plan because they live with their mothers; they go loitering and come back in the evening. They don't know if the baby has eaten or not. So it's their mothers who struggle with the babies … Some don't have someone to leave their children with at night. So they give the babies Piriton [a sedating antihistamine] at night and they go … I think it's hard giving birth when you are young because you haven't had fun … You find a girl disappears even for a week then comes back, and she is not questioned. (FGD, Young Mothers, Korogocho)

Because young mothers are also often single mothers with no other source of livelihood, they get involved in commercial sex work to make ends meet, so ‘most of the children who are not healthy belong to young girls in this community since they are more concerned with work’ (KII, TBA, Viwandani). Another key issue related to young motherhood was self‐efficacy and confidence in regard to breastfeeding.

They mostly have fear of breastfeeding, this fear makes them not breastfeed because of fear of being seen breastfeeding since they are still young, and to remove the breast in public will be difficult. (KII, Youth Leader, Korogocho)

They reported not knowing how to attach the baby to the breast: ‘there is an angle babies are placed when breastfeeding and those young ones don't know’ (FGD, Village Elders, Viwandani). ‘Like the one who gave birth when she was 14 years, the breast became sick, and she gave the child infant formula … Even mine used to breastfeed on one breast, and the other one had more milk hence became sick’ (KII, Women Leader, Viwandani).

Additionally, breastfeeding practices among girls were said to be influenced by peer pressure: ‘The others will tell her that the child is big and ask why she is still breastfeeding … So ‘the duration of breastfeeding is maybe one year and most of them don't want to exceed’ (KII, TBA, Korogocho).

Given all these factors, many girls opt not to initiate breastfeeding, or do not breastfeed optimally as ‘a girl's child eats what the girl eats. If she buys chips then the baby will eat those chips’ (FGD, Young Mothers, Korogocho).

Infant feeding practices among girls were said to be strongly informed by their own mothers who may not be well informed about optimal breastfeeding practices. However, some parents were said to reinforce breastfeeding among these girls: ‘The parent is the one to insist for her to breastfeed … Most of the work is the parent's responsibility’ (FGD, Village Elders, Korogocho).

### Poor social and professional support

#### Poor social support

Lack of adequate social support at the household level was considered a key barrier to optimal breastfeeding practices. Some men considered the issue of breastfeeding as a ‘women's issue’, yet they were said to be the main decision makers with regard to maternal and child care and avail money to access health care. They sometimes have strong opinions that go against optimal breastfeeding practices and ‘women would listen to their husbands before anybody else.’ Some husbands were said to be unsupportive to the women when drunk because they would quarrel a lot. Others compete for attention with the baby and sometimes ask the mothers to stop breastfeeding for this reason.

Mothers have no time to rest and recover from delivery, and were often stressed because of too much work in the house. Unlike their rural counterparts who may have relatives stepping in to help, or the richer women in non‐slum settings who may afford paid help, women in the slums often have no one to help with daily chores. Some women start on the chores immediately after delivery.

Most women here wake up and work and forget they have a baby. By the time they remember they have a baby, there is no milk in the breast. So mostly, those who have milk are those who have maids so the maids work while they relax … There are men who don't give their wives time to rest … there are some men who don't care, as long as there is the woman in the house, they don't get tired, they want sex every time. (FGD, Community Health Workers, Korogocho)

#### Poor professional support

It was reported that many women do not access maternity care services at standard health facilities. They often deliver at home or at suboptimal clinics and therefore do not access the counselling and support on breastfeeding. Additionally, there were reports that health care professionals are often too busy to offer breastfeeding counselling to mothers.

Now you'll find the problem which is here, is that the child is born and after one month it is given these adults’ foods. That is one problem and it is as if the mother doesn't know … Most children are born just here at home … The mother didn't even go to the clinic, she was not taught about how to take care of the child, she doesn't know if she is (HIV) positive or negative. (FGD, Village Elders, Viwandani)

### Poor knowledge, myths and misconceptions

While most people were said to hold the practice of feeding babies on colostrum in high regard, some people delay initiation and others do not give colostrum to the baby because they do not know of its importance. While ‘some say it is dirt’, some people believe it is just water, and would therefore give babies pre‐lacteal foods as they await the ‘real’ milk. According to the respondents, among some social groups such as the Sukuma people who migrated to the slums from Tanzania, colostrum is not given to the baby as it is considered to cause diseases such as leprosy or eye disease.

With regard to the period of exclusive breastfeeding, some people believe a little water and sugar/glucose and/or salt or commercially prepared mixture of water (gripe water) is good for the baby as this would protect the baby from stomach problems. Some community health/social workers also hold this belief.

I breastfed him. I breastfed him when I got out of the hospital and when I noticed that he had stomach upsets I introduced him to water. (IDI, Young Mother, Viwandani)

I cannot be clear about age but water should be introduced when the child is about two months. The mother should give about two spoons of water at this age, a requirement not practiced by people in this community; when we do our rounds (as community health workers), you come across a mother with a whole bottle of water alongside that for milk to be given to the child. (KII, TBA who doubles as a community health worker, Viwandani)

Some people believe that breast milk alone is not adequate for the baby, especially boys. They cry excessively due to hunger and once they are given food, they stop crying. The size of the baby at birth was also considered important; children born larger than normal were thought to need more than breast milk, so ‘you are forced to give other foods like porridge even before one month’ (FGD, Older Mothers, Korogocho). Older mothers also reported that if you exclusively breastfeed the baby for 6 months, the baby will have problems when initiating foods, so it is better to initiate other foods slowly even before the end of the first 6 months.

I used to give only water … two spoons only. I thought the baby was never having enough from the breast, because the child used to cry often. (IDI, Young Mother, Korogocho)

Religious teachings sometimes determine breastfeeding practices. For example, according to the Quran, a child should be breastfed for exactly 2 years, but one should not exceed the 2 years even by 1 day. There were also beliefs that if a child breastfeeds for too long, they would become foolish, while others believe that if a child has delayed walking, if you stop breastfeeding the baby will start walking. As noted earlier, others considered breastfeeding as a way of family planning and to conceive they must stop breastfeeding first. There were also beliefs that if one falls pregnant, she should not continue breastfeeding as this will have negative effects on the breastfeeding baby. Other people, particularly from the Luo community, believed that one should not continue breastfeeding if she has sex with a man who is not the father of the child as this will drastically affect the breastfeeding child as the child may get ‘chira’ (cultural disease in the Luo community) and become thin. This is particularly important given that commercial sex work was said to be common.

Other beliefs and myths included that if one breastfeeds in public, a person with an ‘evil eye’ may look at them and this may cause the breasts to have sores; if one quarrels with her husband or a neighbour, one cannot breastfeed until a cultural ritual (Manyasi in the Luhya community) to cleanse her and the baby is performed; and that breastfeeding a baby after a gap of a whole day may cause illness to the baby such as diarrhoea.

### HIV

#### Fear of mother‐to‐child transmission

HIV, which is prevalent in the study setting at 12% among adults (Madise *et al*. 2012), was also considered an important factor affecting breastfeeding practices. Some people who are HIV positive or those who do not know their status fear breastfeeding due to risk of mother‐to‐child transmission. There were mixed views among the various respondents regarding how HIV‐positive women should breastfeed, with some suggesting that an HIV‐positive woman should not breastfeed at all; others that she should exclusively breastfeed for 6 months or 1 year. Most respondents believed that an HIV‐positive mother should avoid mixed feeding for fear of infecting the child; even if the child is breastfed exclusively for 6 months, breastfeeding should stop completely when foods are introduced.

This time round, they tell us that we should breastfeed for six months and even up to one year without giving anything … even without giving water … if a child is given foods or water, there are sores that appear in the baby's mouth or throat. This is the reason why we should give breast milk only … I breastfed exclusively for seven months. (IDI, HIV‐Positive Mother, Viwandani)

If you decide it's food, you give it [the baby] food, if you decide it's breast milk you give it breast for six months; do not mix; if food, food only without breast … (otherwise) you will infect the child. (FGD, Young Mothers, Korogocho)

While some people may be informed about the policy on extended breastfeeding for 1 year for HIV‐positive women, there is fear of extended breastfeeding beyond 6 months as the child may have developed teeth as 6 months, and may bite the mother, leading to infection of the baby with HIV as an HIV‐positive woman indicates:

[A baby should breastfeed] for six months exclusively then continue till one year but if one is able she can do exclusive breastfeeding for one year. However, here in Korogocho you will find that after the six month, the baby has developed teeth therefore will bite the mother if she doesn't have the milk and contract HIV. (IDI, HIV‐Positive Mother, Korogocho)

#### Stigma

Some people associate exclusive breastfeeding with HIV, as previous counselling regarding breastfeeding by health care workers has emphasised strict exclusive breastfeeding followed by rapid weaning for HIV‐positive women. Some people were therefore said to avoid exclusive breastfeeding even those who are HIV positive so that they are not associated with HIV, and some were said to brush off advice on exclusive breastfeeding by saying they are not HIV positive:

If I go and tell my friend not to give the baby food but breastfeed she will answer me back rudely and claim I think she has HIV. So it's good people like you to tell them … Some people believe those who breastfeed exclusively for six months are the mothers who are HIV positive. (FGD, Young Mothers, Korogocho)

### Unintended pregnancies

The respondents indicated that some children are not breastfed optimally due to untimely unintended pregnancies. Women were said to often conceive when the breastfeeding child was still young while many people believe that one should not breastfeed while pregnant as this would affect the breastfeeding baby including causing death to the baby.

What I have heard is that if a mother conceives again when the baby is still young, say if the baby is one year and the mother conceives, she will not continue breastfeeding this other one. They say, according to traditions, the baby which is breastfeeding will die. So when they realize they are pregnant even if the other one is six months, they stop. You know there are those who conceive after three months? (KII, Health Professional, Viwandani)

There were many beliefs associated with family planning which may be the reason for unintended pregnancies. Some people believe that one cannot conceive while breastfeeding, hence they do not use modern contraceptives when breastfeeding and they often end up with unplanned pregnancies. Conversely, if they want to conceive, they stop breastfeeding:

Some mothers use breastfeeding as a method of family planning, and there are some men who just want children. Maybe you gave birth to a baby boy, and he wants a girl. So you are forced to stop breastfeeding the baby so that you can conceive again. (FGD, CHWs, Korogocho)

Other people believe that modern family planning methods would harm them, e.g. reduce milk production, change the colour of the milk, reduce sexual urge or make people become fat. Some believe that family planning methods may harm the baby causing short‐term (illness) and long‐term damage (infertility) to the baby as the baby ‘can suckle it (contraceptive) from the breasts’.

Say when using family planning drugs, and if the baby becomes big it can suckle that from the breasts, and it becomes sick. We just say if the baby reaches two years you should not breastfeed because that family planning drug can harm your child. Some say that a child will not bear children when it grows up, if it has reached one year and suckles your breasts which have the drug. We say your child will not bear children. (KII, Religious Leader, Korogocho)

## Discussion

This study has examined breastfeeding practices and the factors affecting actualisation of WHO recommendations for breastfeeding in urban poor settings in Nairobi, Kenya. An earlier study quantitatively identified various factors that affect breastfeeding and other infant feeding practices in the study setting including child's sex, perceived size at birth; mother's marital status, ethnicity and education level; whether pregnancy was desired or not; and place of delivery (Kimani‐Murage *et al*. 2011), but it did not explore in detail the socio‐cultural barriers to optimal breastfeeding which are described in the current study. The narratives in the current study more or less explain the factors identified quantitatively. Much as the respondents in this study showed awareness of optimal infant and young child feeding practices, the practical realities were that it was not always possible to adhere to good practices because of social‐cultural, socio‐economic and socio‐demographic factors, the prevalence of which have been documented in other studies (African Population and Health Research Center 2002a; Fotso *et al*. 2009; Ndugwa *et al*. 2011; Madise *et al*. 2012; Beguy *et al*. 2013a, 2013b; Mberu *et al*. 2014).

Ecological theories and conceptual frameworks are important in guiding interpretation of research findings and effective planning and implementation of public health interventions (Hector *et al*. 2005; McLaren & Hawe 2005). A growing body of evidence shows the importance of context particularly social‐cultural environment in maternal and child nutrition (Morisky *et al*. 2002; Fikree *et al*. 2005; Dykes & Hall‐Moran 2009). McLaren & Hawe (2005) illustrate how an individual interacts with his environment made of various systems including microsystem (e.g. family, school, peers), mesosystem (linkages or overlaps between settings in which the individual participates, e.g. the overlap between family time and work time when one works overtime), exosystem (e.g. local politics, neighbours, mass media, social services) and macrosystem (including attitudes and cultural ideologies) (McLaren & Hawe 2005). It is therefore important to study the people's environment when designing health interventions for them. We have proposed a draft conceptual framework (Fig. 2) of factors that affect breastfeeding practices in urban poor settings at three levels (individual, group and societal), modelled around the framework by Hector *et al*. (2005). Like that model, our model illustrates how interactions between an individual with his environment including the home/family environment, health services, work environment, community environment, and the political, social, economic and cultural environment determine the ultimate breastfeeding practices. Although Hector et al.'s model was developed based on experiences of high‐income countries, findings of the current study seem to fit well with the model. However, the specific factors that fit in the various levels in our model are probably quite different from the specific factors that would fit in the levels based on data from high‐income countries. Additionally, factors affecting breastfeeding in urban poor settings are more complex, as it seems that some factors cross over the different levels, and it is difficult to pin the factors to only one specific level. Such a factor is, for example, alcoholism that fits all levels: at the individual level where some mothers were said to be alcoholic; at the group level as many men (spouses) were said to be alcoholic, leading to a non‐conducive environment for breastfeeding in the household as the men are not supportive of the breastfeeding mothers; and also at the community level as the issue of alcoholism was said to be a societal problem common in the study setting (Ayah *et al*. 2013; Oti *et al*. 2013). Our framework highlights the complex nature of factors that affect breastfeeding in urban poor settings, and echoes the conclusions by Dykes & Hall‐Moran (2009) regarding challenges of implementing the global strategy for infant and young child feeding, given the context‐specific factors that influence feeding practices (Dykes & Hall‐Moran 2009). The framework may guide generation of hypothesis regarding factors affecting breastfeeding in urban poor settings that can be tested in the future, and guide interventions to improve breastfeeding practices and consequently child nutrition in urban poor settings.

Poverty, livelihood and living conditions featured very prominently in this study as key factors influencing breastfeeding practices among urban poor women. Poverty and poor living conditions and consequent risky behaviours have been reported as prevalent in the study setting in quantitative studies (African Population and Health Research Center 2002a,b; Zulu *et al*. 2002; Oti *et al*. 2013, Mberu *et al*. 2014). In the previous study cited above (Kimani‐Murage *et al*. 2011), respondents indicated that one key reason for not breastfeeding optimally was inadequate breast milk. In the current study, respondents associated inadequate breast milk and hence early initiation of complementary foods to lack of adequate food for the breastfeeding mothers to eat. This has also been found in other studies in the slums in India (Roy *et al*. 2009). Household food insecurity is rampant in the study area, as 85% of all households are food insecure, while half (50%) are severely food insecure (Kimani‐Murage *et al*. 2014). While health education needs to unravel the myths relating to inadequate food for the mother to eat and breast milk production, which is usually a hormonal process, there is a need to address the issue of food insecurity in the study setting to ensure good nutritional status and health for the mothers, which may result in good child feeding and general care (United Nations Children's Fund 1990; Engle *et al*. 1999; Bhutta *et al*. 2013). However, little evidence exists on the effect of food security on infant feeding practices (Saha *et al*. 2008), hence more research is needed.

In the context of rampant poverty in the study settings (Mberu *et al*. 2014), livelihoods and nature of work for the breastfeeding mother were considered key in determining breastfeeding practices, affecting both duration of exclusive breastfeeding and duration and quality of any breastfeeding. Mother's occupation has also been identified as a factor influencing breastfeeding practices in other settings (Mahgoub *et al*. 2002; Sasaki *et al*. 2010). Privileges taken for granted in the formal employment sector such as the 90 days paid maternity leave are non‐existent among the urban poor who depend on casual employment. Mothers have to resume work shortly after giving birth, especially because they need money to support themselves and their families. The residents mostly work as casual labourers in neighbouring factories or as domestic workers in nearby middle‐income communities. They earn a limited income and are therefore not able to keep savings to use during times of need such as after delivery. Expressing breast milk, which would partly help deal with the problem, was uncommon or unacceptable in the study settings. There is a need for awareness regarding how to manage breastfeeding even when the mother is separated from the baby, including expressing, and safely storing the expressed breast milk. Revision of labour laws to accommodate casual labourers and social protection measures such as cash transfers for breastfeeding mothers in urban poor settings are warranted, given that child nutrition and therefore breastfeeding is a human right. Duration of maternity leave has been associated with breastfeeding practices although such evidence is more common in more developed countries (Hawkins *et al*. 2007; Staehelin *et al*. 2007; Calnen 2010; Aikawa *et al*. 2012). There is also limited evidence of the effectiveness of cash transfers on breastfeeding practices (Bassani *et al*. 2013). There is therefore a need for more research on the effectiveness of these social protection measures on breastfeeding practices.

Early pregnancies, closely linked to single motherhood in the study community, were considered major determinants of breastfeeding practices. The young mothers in the current study were said not to know how to breastfeed, not to have self‐confidence in breastfeeding, or to care about their body image hence not to want to breastfeed at all, as they often engaged in sex for survival. They were said to have very poor child care practices. From a census of all births that happened in the study area over a period of 5 years from 2006 to 2010, evidence indicates that slightly over half of the births are by mothers under 25 years (Kimani‐Murage *et al*. 2011). Previous studies in the study setting have indicated prevalence of early sexual debut and early motherhood (Zulu *et al*. 2002; Ndugwa *et al*. 2011; Beguy *et al*. 2013a, 2013b; Marston *et al*. 2013), with a substantial proportion (close to 10%) of adolescents being sexually active before 15 years (Ndugwa *et al*. 2011), and 15% of adolescents 15–19 years of age ever having a child (Beguy *et al*. 2013a, 2013b). Similar findings have been reported in other urban settings in SSA (Meekers & Ahmed 2000). The high level of early sexual activity and early motherhood (Zulu *et al*. 2002; Beguy *et al*. 2013a, 2013b; Marston *et al*. 2013) is occasioned by the rampant poverty, as reported in this study, and as evidence indicates that deprivation is a key factor in risky sexual behaviour and transactional sex in Nairobi slums (Greif 2012). The area is plagued with a high prevalence of unintended pregnancies, with more than 40% of pregnancies among adolescents being unintended yet contraceptive use among the adolescents is limited (Beguy *et al*. 2013a, 2013b; Beguy *et al*. 2014). The findings with regard to the relationship between family planning, unplanned pregnancies, breastfeeding and the health of the child are therefore interesting. There is a need to ensure access to information and services on contraceptives for the wider community and also through youth‐friendly reproductive health programmes. Further, there is a need for early education on maternal and child nutrition, and provision of access to readily available professional support to young breastfeeding mothers, possibly through breastfeeding support programmes targeted at young mothers.

HIV/AIDS prevalence in Nairobi's slums is 12% (Madise *et al*. 2012), approximately twice the national prevalence and the prevalence for Nairobi as a whole, and was considered a major determinant of breastfeeding practices. There is mixed understanding regarding breastfeeding for HIV‐positive women due to the confusion caused by the ever changing recommendations regarding breastfeeding for HIV‐positive women, as also documented elsewhere in SSA (Chisenga *et al*. 2011). There is also stigma associated with HIV/AIDS, which affects practice for women living with HIV and the uninfected women. For example, because exclusive breastfeeding is associated with HIV‐positive women, HIV‐positive women prefer to hide under mixed feeding like most other women do, while the HIV‐negative women do not want to exclusively breastfeed so as not to be suspected as being HIV positive. HIV stigma related to breastfeeding has also been reported in other urban areas in SSA (Ostergaard & Bula 2010; Chisenga *et al*. 2011). There is a need to ensure correct and consistent messaging on breastfeeding for HIV‐positive women through frequent awareness of health care workers and the community at large on the recommended practices.

Poor social and professional support was reported as key factor in determining breastfeeding practices in the study setting. At the household level, support comes from the spouse, yet in this study men were commonly said to be ignorant of this issue. At the same time, they were said to be the main decision makers with regard to maternal and child care issues. However, though questions on men involvement were included in the interviews, men were not directly targeted specifically to get their views on the issues of breastfeeding. It is therefore difficult to know their true views. Nevertheless, while it is recommended that studies in the future also target interviews with men specifically to get their true perceptions, based on the views of other respondents, it may be useful to create awareness among the men regarding optimal breastfeeding practices. It is generally assumed that the mothers who attend and deliver at health facilities would get information at the health facility, but the report that health care professionals are often too busy to offer breastfeeding counselling to mothers is disturbing and may explain some of the misconceptions and poor practices seen in this community. Such misconceptions could be addressed through provision of access to readily available professional support to all mothers possibly through community‐based breastfeeding support programmes and at the maternal and child health clinics.

The strength of this study is the variety of respondents who covered a depth of representatives from the community; it included women of different religions, ages and ethnicities. It also included community leadership. The opinions expressed therefore can be taken as an accurate reflection of community knowledge, beliefs and practices. The study identified many important issues and difficulties the mothers face that need to be addressed in order to improve infant feeding practices. However, a negative belief is not easily replaced by a viable plausible option. Examples of mothers who have successfully breastfed their babies exclusively within the same community may be one way of convincing people that it can work. On the positive side, there is good knowledge on some of the necessary practices. So the intervention process will need to be tailored to this and find ways of overcoming obstacles/hindrances. There is a need to bring support close to mothers through interventions such as home‐based counselling by community health workers and formation of mother support groups. Counselling that includes practical skills as well as sensitising household members about supporting and accommodating the breastfeeding mother may be a first step in this process. But for urban slum settings, approaches aimed at improving breastfeeding practices must consider the wider ecological setting in order to be successful. Future studies need to also specifically target perceptions of men, given that they were considered key in decision making for IYCN.

In conclusion, this study finds that a set of factors including misconceptions, socio‐economic and socio‐cultural factors is perceived to shape breastfeeding behaviours among urban poor mothers in Nairobi. The study reveals that women in urban poor settings face an extremely complex situation with regard to breastfeeding due to multiple challenges and risk behaviours often dictated to them by their circumstances and context. The reality is that they are unlikely to change their circumstances. While interventions focusing on individual behaviour change may dispel some of the myths and misconceptions regarding breastfeeding, macro‐level policies and interventions are needed. Such macro‐level policies and interventions may include revision of labour laws regarding maternity leave in favour of casual labourers, provision of social protection measures for urban poor breastfeeding mothers, adequate child care facilities, support for mothers expressing breast milk, provision of youth‐friendly family planning services and incorporation of MIYCN training in school curricula. There is also need for community‐wide awareness of the need to support breastfeeding mothers within their communities. Family support is particularly important and should be promoted alongside male involvement in maternal and child health issues as it was evident from the interviews that men shy away from involvement in these issues. Given the importance of nutrition in the first 1000 days of life to the child's health, growth, development and survival, and future economic productivity, interventions targeted at child health, survival and well‐being need to start early in life. Failure of intervening early may lead to long‐term economic consequences and increased health burden.

## Sources of funding

This study was funded by the Wellcome Trust (Grant No. 097146/Z/11/Z). This research was also made possible through the generous core funding for APHRC by the William and Flora Hewlett Foundation (Grant No. 2009‐40510) and the Swedish International Cooperation Agency (SIDA) (Grant No. 2011‐001578). PG is supported by a British Academy Mid‐Career Fellowship (Ref: MD120048).

## Conflicts of interest

The authors declare that they have no conflicts of interest.

## Contributions

EWK‐M: Designed the study, coded the data, analysed the data, wrote the manuscript and approved the manuscript for submission. PG: Designed the study, analysed the data, guided the writing of the manuscript, reviewed the manuscript and approved the manuscript for submission. FW: Designed the study, supervised field work, analysed the data, reviewed the manuscript and approved the manuscript for submission. MW: Participated in data collection, coded the data, analysed the data, reviewed the manuscript and approved the manuscript for submission. CK, ACE, RNM, SAN and NJM: Designed the study, reviewed the manuscript and approved the manuscript for submission.

## References

[mcn12161-bib-0001] Abuya B.A. , Ciera J. & Kimani‐Murage E. (2012) Effect of mother's education on child's nutritional status in the slums of Nairobi. BMC Pediatrics 12, 80. 22721431 10.1186/1471-2431-12-80 PMC3444953

[mcn12161-bib-0002] Aikawa T. , Pavadhgul P. , Chongsuwat R. , Sawasdivorn S. & Boonshuyar C. (2012) Maternal return to paid work and breastfeeding practices in Bangkok, Thailand. Asia‐Pacific Journal of Public Health doi: 10.1177/1010539511419647. 22815310

[mcn12161-bib-0003] Akter S. & Rahman M.M. (2010) The determinants of early cessation of breastfeeding in Bangladesh. World Health and Population 11, 5–12. 10.12927/whp.2010.21722 20739835

[mcn12161-bib-0004] African Population and Health Research Center (APHRC) (2002a) Population and Health Dynamics in Nairobi Informal Settlements, APHRC: Nairobi.

[mcn12161-bib-0005] African Population and Health Research Center (APHRC) (2002b) Health and Livelihood Needs Of Residents of Informal Settlements on Nairobi City, *Occasional Study Report 1*, APHRC: Nairobi.

[mcn12161-bib-0006] Ayah R. , Joshi M.D. , Wanjiru R. , Njau E.K. , Otieno C.F. , Njeru E.K. *et al* (2013) A population‐based survey of prevalence of diabetes and correlates in an urban slum community in Nairobi, Kenya. BMC Public Health 13, 371. 23601475 10.1186/1471-2458-13-371 PMC3641964

[mcn12161-bib-0007] Bassani D.G. , Arora P. , Wazny K. , Gaffey M.F. , Lenters L. & Bhutta Z.A. (2013) Financial incentives and coverage of child health interventions: a systematic review and meta‐analysis. BMC Public Health 13 (Suppl. 3), S30. 24564520 10.1186/1471-2458-13-S3-S30 PMC3847540

[mcn12161-bib-0008] Beguy D. , Mumah J. , Wawire S. , Muindi K. , Gottschalk L. & Kabiru C.W. (2013a) Status report on the sexual and reproductive health of adolescents living in urban slums in Kenya. *STEP UP Technical Working Paper* . Nairobi: APHRC.

[mcn12161-bib-0009] Beguy D. , Ndugwa R. & Kabiru C.W. (2013b) Entry into motherhood among adolescent girls in two informal settlements in Nairobi, Kenya. Journal of Biosocial Science 45, 721–742. 23688912 10.1017/S0021932013000199 PMC3785175

[mcn12161-bib-0010] Beguy D. , Mumah J. & Gottschalk L. (2014) Unintended pregnancies among young women living in urban slums: evidence from a prospective study in Nairobi city, Kenya. PLoS ONE 9, e101034. 25080352 10.1371/journal.pone.0101034 PMC4117474

[mcn12161-bib-0011] Bhutta Z.A. , Das J.K. , Rizvi A. , Gaffey M.F. , Walker N. , Horton S. *et al* (2013) Evidence‐based interventions for improvement of maternal and child nutrition: what can be done and at what cost? Lancet 382, 452–477. 23746776 10.1016/S0140-6736(13)60996-4

[mcn12161-bib-0012] Calnen G. (2010) The impact of maternity leave on breastfeeding rates. Breastfeeding Medicine 5, 233–234. 20942708 10.1089/bfm.2010.0064

[mcn12161-bib-0013] Chisenga M. , Siame J. , Baisley K. , Kasonka L. & Filteau S. (2011) Determinants of infant feeding choices by Zambian mothers: a mixed quantitative and qualitative study. Maternal and Child Nutrition 7, 148–159. 21410882 10.1111/j.1740-8709.2010.00264.x PMC6860822

[mcn12161-bib-0014] Dykes F. & Hall‐Moran V. (eds) (2009) Infant and Young Child Feeding: Challenges to Implementing a Global Strategy, Wiley‐Blackwell: Oxford.

[mcn12161-bib-0015] Emina J. , Beguy D. , Zulu E.M. , Ezeh A.C. , Muindi K. , Elung'ata P. *et al* (2011) Monitoring of health and demographic outcomes in poor urban settlements: evidence from the Nairobi Urban Health and Demographic Surveillance System. Journal of Urban Health: Bulletin of the New York Academy of Medicine 88 (Suppl. 2), S200–S218. 21713553 10.1007/s11524-011-9594-1 PMC3132229

[mcn12161-bib-0016] Engle P.L. , Menon P. & Haddad L. (1999) Care and nutrition: concepts and measurement. World Development 27, 1309–1337.

[mcn12161-bib-0017] Fikree F.F. , Ali T.S. , Durocher J.M. & Rahbar M.H. (2005) Newborn care practices in low socioeconomic settlements of Karachi, Pakistan. Social Science and Medicine 60, 911–921. 15589663 10.1016/j.socscimed.2004.06.034

[mcn12161-bib-0018] Fotso J.‐C. , Ezeh A. , Madise N. , Ziraba A. & Ogollah R. (2009) What does access to maternal care mean among the urban poor? Factors associated with use of appropriate maternal health services in the slum settlements of Nairobi, Kenya. Maternal and Child Health Journal 13, 130–137. 18297380 10.1007/s10995-008-0326-4

[mcn12161-bib-0019] Fotso J.C. , Madise N. , Baschieri A. , Cleland J. , Zulu E. , Mutua M.K. *et al* (2012) Child growth in urban deprived settings: does household poverty status matter? At which stage of child development? Health and Place 18, 375–384. 22221652 10.1016/j.healthplace.2011.12.003 PMC3701841

[mcn12161-bib-0020] Gareth J. , Richard W.S. , Robert E.B. , Zulfiqar A.B. & Saul S.M. (2003) How many child deaths can we prevent this year? Lancet 362, 65–71. 12853204 10.1016/S0140-6736(03)13811-1

[mcn12161-bib-0021] Grantham‐McGregor S. , Cheung Y.B. , Cueto S. , Glewwe P. , Richter L. & Strupp B. (2007) Developmental potential in the first 5 years for children in developing countries. Lancet 369, 60–70. 17208643 10.1016/S0140-6736(07)60032-4 PMC2270351

[mcn12161-bib-0022] Greif M.J. (2012) Housing, medical, and food deprivation in poor urban contexts: implications for multiple sexual partnerships and transactional sex in Nairobi's slums. Health and Place 18, 400–407. 22257740 10.1016/j.healthplace.2011.12.008

[mcn12161-bib-0023] Hawkins S.S. , Griffiths L.J. , Dezateux C. , Law C. & Millennium Cohort Study Child Health Group (2007) The impact of maternal employment on breast‐feeding duration in the UK Millennium Cohort Study. Public Health Nutrition 10, 891–896. 17381907 10.1017/S1368980007226096

[mcn12161-bib-0024] Hector D. , King L. , Webb K. & Heywood P. (2005) Factors affecting breastfeeding practices. Applying a conceptual framework. New South Wales Public Health Bulletin 16, 52–55. 16106273 10.1071/nb05013

[mcn12161-bib-0025] Ijumba P. , Doherty T. , Jackson D. , Tomlinson M. , Sanders D. & Persson L.A. (2014) Social circumstances that drive early introduction of formula milk: an exploratory qualitative study in a peri‐urban South African community. Maternal and Child Nutrition 10, 102–111. 23230962 10.1111/mcn.12012 PMC6860256

[mcn12161-bib-0026] Isolauri E., Martorell R. & Eriksson J.G. (eds.) 2011 *Importance of nutrition during the first 1000 days of life* .

[mcn12161-bib-0027] Kenya National Bureau of Statistics & ICF Macro (2009) Kenya Demographic and Health Survey 2008–09, KNBS and ICF Macro: Calverton, MD.

[mcn12161-bib-0028] Kerac M. , Blencowe H. , Grijalva‐Eternod C. , McGrath M. , Shoham J. , Cole T.J. *et al* (2011) Prevalence of wasting among under 6‐month‐old infants in developing countries and implications of new case definitions using WHO growth standards: a secondary data analysis. Archives of Disease in Childhood 96, 1008–1013. 21288999 10.1136/adc.2010.191882 PMC3195296

[mcn12161-bib-0029] Kimani‐Murage E. , Madise N.J. , Fotso J.‐C. , Kyobutungi C. , Mutua K. , Gitau T. *et al* (2011) Patterns and determinants of breastfeeding and complementary feeding practices in urban informal settlements, Nairobi Kenya. BMC Public Health 11, 396. 21615957 10.1186/1471-2458-11-396 PMC3118248

[mcn12161-bib-0030] Kimani‐Murage E.W. & Ngindu A.M. (2007) Quality of water the slum dwellers use: the case of a Kenyan slum. Journal of Urban Health: Bulletin of the New York Academy of Medicine 84, 829–838. 17551841 10.1007/s11524-007-9199-x PMC2134844

[mcn12161-bib-0031] Kimani‐Murage E.W. , Schofield L. , Wekesah F. , Mohamed S. , Mberu B. , Ettarh R. *et al* (2014) Vulnerability to food insecurity in urban slums: experiences from Nairobi, Kenya. Journal of Urban Health: Bulletin of the New York Academy of Medicine doi: 10.1007/s11524-014-9894-3. PMC4242851 25172616

[mcn12161-bib-0032] Kramer M.S. & Kakuma R. (2004) The optimal duration of exclusive breastfeeding: a systematic review. Advances in Experimental Medicine and Biology 554, 63–77. 15384567 10.1007/978-1-4757-4242-8_7

[mcn12161-bib-0033] Lacey A. & Luff D. 2001 Trent focus for research and development in primary health care: an introduction to qualitative analysis. Trent Focus.

[mcn12161-bib-0034] Lanigan J. & Singhal A. (2009) Early nutrition and long‐term health: a practical approach. The Proceedings of the Nutrition Society 68, 422–429. 19698202 10.1017/S002966510999019X

[mcn12161-bib-0035] Lauer J. , Betran A. , Victora C. , De Onis M. & Barros A. (2004) Breastfeeding patterns and exposure to suboptimal breastfeeding among children in developing countries: review and analysis of nationally representative surveys. BMC Medicine 2, 26. 15230974 10.1186/1741-7015-2-26 PMC455698

[mcn12161-bib-0036] Madise N.J. , Ziraba A.K. , Inungu J. , Khamadi S.A. , Ezeh A. , Zulu E.M. *et al* (2012) Are slum dwellers at heightened risk of HIV infection than other urban residents? Evidence from population‐based HIV prevalence surveys in Kenya. Health and Place 18, 1144–1152. 22591621 10.1016/j.healthplace.2012.04.003 PMC3427858

[mcn12161-bib-0037] Mahgoub S.E.O. , Bandeke T. & Nnyepia M. (2002) Breastfeeding in Botswana: practices, attitudes, patterns, and the socio‐cultural factors affecting them. Journal of Tropical Pediatrics 48, 195–199. 12200978 10.1093/tropej/48.4.195

[mcn12161-bib-0038] Marston M. , Beguy D. , Kabiru C. & Cleland J. (2013) Predictors of sexual debut among young adolescents in Nairobi's informal settlements. International Perspectives on Sexual and Reproductive Health 39, 22–31. 23584465 10.1363/3902213 PMC4101799

[mcn12161-bib-0039] Matsuyama A. , Karama M. , Tanaka J. & Kaneko S. (2013) Perceptions of caregivers about health and nutritional problems and feeding practices of infants: a qualitative study on exclusive breast‐feeding in Kwale, Kenya. BMC Public Health 13, 525. 23721248 10.1186/1471-2458-13-525 PMC3681582

[mcn12161-bib-0040] Mberu B.U. , Ciera J.M. , Elungata P. & Ezeh A.C. (2014) Patterns and determinants of poverty transitions among poor urban households in Nairobi. African Development Review 26, 172–185.

[mcn12161-bib-0041] McLaren L. & Hawe P. (2005) Ecological perspectives in health research. Journal of Epidemiology and Community Health 59, 6–14. 15598720 10.1136/jech.2003.018044 PMC1763359

[mcn12161-bib-0042] Meekers D. & Ahmed G. (2000) Contemporary patterns of adolescent sexuality in urban Botswana. Journal of Biosocial Science 32, 467–485. 11075640 10.1017/s0021932000004673

[mcn12161-bib-0043] Morisky D.E. , Snehendu B.K. , Chaudrhry A.S. , Chen K.R. , Shaheen M. & Chickering K. (2002) Breastfeeding practices in Pakistan. Pakistan Journal of Nutrition 1, 137–142.

[mcn12161-bib-0044] Ndugwa R.P. , Kabiru C.W. , Cleland J. , Beguy D. , Egondi T. , Zulu E.M. *et al* (2011) Adolescent problem behavior in Nairobi's informal settlements: applying problem behavior theory in sub‐Saharan Africa. Journal of Urban Health: Bulletin of the New York Academy of Medicine 88 (Suppl. 2), S298–S317. 20499192 10.1007/s11524-010-9462-4 PMC3132234

[mcn12161-bib-0045] Oddy W.H. , Kendall G.E. , Blair E. , De Klerk N.H. , Stanley F.J. , Landau L.I. *et al* (2003) Breast feeding and cognitive development in childhood: a prospective birth cohort study. Paediatric and Perinatal Epidemiology 17, 81–90. 12562475 10.1046/j.1365-3016.2003.00464.x

[mcn12161-bib-0046] Olack B. , Burke H. , Cosmas L. , Bamrah S. , Dooling K. , Feikin D.R. *et al* (2011) Nutritional status of under‐five children living in an informal urban settlement in Nairobi, Kenya. Journal of Health, Population, and Nutrition 29, 357–363. 10.3329/jhpn.v29i4.8451 PMC3190366 21957674

[mcn12161-bib-0047] Ostergaard L.R. & Bula A. (2010) They call our children ‘Nevirapine babies?’: a qualitative study about exclusive breastfeeding among HIV positive mothers in Malawi. African Journal of Reproductive Health 14, 213–222. 21495616

[mcn12161-bib-0048] Oti S.O. , Van De Vijver S.J. , Agyemang C. & Kyobutungi C. (2013) The magnitude of diabetes and its association with obesity in the slums of Nairobi, Kenya: results from a cross‐sectional survey. Tropical Medicine and International Health 18, 1520–1530. 24118454 10.1111/tmi.12200

[mcn12161-bib-0049] Pan American Health Organization (2003) Guiding Principles for Complementary Feeding of the Breastfed Child, WHO: Washington, DC.

[mcn12161-bib-0050] Pascale K.N.A. , Laure N.J. & Enyong O.J. (2007) Factors associated with breast feeding as well as the nutritional status of infants (0–12) months: an epidemiological study in Yaounde, Cameroon. Pakistan Journal of Nutrition 6, 259–263.

[mcn12161-bib-0051] Patel A. , Badhoniya N. , Khadse S. , Senarath U. , Agho K.E. & Dibley M.J. (2010) Infant and young child feeding indicators and determinants of poor feeding practices in India: secondary data analysis of National Family Health Survey 2005–06. Food and Nutrition Bulletin 31, 314–333. 20707236 10.1177/156482651003100221

[mcn12161-bib-0052] Roy S. , Dasgupta A. & Pal B. (2009) Feeding practices of children in an urban slum of Kolkata. Indian Journal of Community Medicine: Official Publication of Indian Association of Preventive & Social Medicine 34, 362–363. 20165637 10.4103/0970-0218.58402 PMC2822204

[mcn12161-bib-0053] Saha K.K. , Frongillo E.A. , Alam D.S. , Arifeen S.E. , Persson L.A. & Rasmussen K.M. (2008) Household food security is associated with infant feeding practices in rural Bangladesh. The Journal of Nutrition 138, 1383–1390. 18567765 10.1093/jn/138.7.1383 PMC2518644

[mcn12161-bib-0054] Sasaki Y. , Ali M. , Kakimoto K. , Saroeun O. , Kanal K. & Kuroiwa C. (2010) Predictors of exclusive breast‐feeding in early infancy: a survey report from Phnom Penh, Cambodia. Journal of Pediatric Nursing 25, 463–469. 21035012 10.1016/j.pedn.2009.04.010

[mcn12161-bib-0055] Setegn T. , Belachew T. , Gerbaba M. , Deribe K. , Deribew A. & Biadgilign S. (2012) Factors associated with exclusive breastfeeding practices among mothers in Goba district, south east Ethiopia: a cross‐sectional study. International Breastfeeding Journal 7, 17. 23186223 10.1186/1746-4358-7-17 PMC3560275

[mcn12161-bib-0056] Staehelin K. , Bertea P.C. & Stutz E.Z. (2007) Length of maternity leave and health of mother and child – a review. International Journal of Public Health 52, 202–209. 18030952 10.1007/s00038-007-5122-1

[mcn12161-bib-0057] United Nations Children's Fund, World Health Organization & the World Bank (2012) UNICEFWHO‐World Bank Joint Child Malnutrition Estimates, UNICEF, WHO and The World Bank: New York, Geneva and Washington, DC.

[mcn12161-bib-0059] United Nations Children's Fund (UNICEF) (1990) Strategies for Improved Nutrition of Children and Women in Developing Countries. A UNICEF Policy Peview, UNICEF: New York.

[mcn12161-bib-0058] United Nations Human Settlements Programme (2008) State of the World's Cities 2010/2011: Bridging the Urban Divide, UNHABITAT: London.

[mcn12161-bib-0060] Victora C.G. , Adair L. , Fall C. , Hallal P.C. , Martorell R. , Richter L. *et al* (2008) Maternal and child undernutrition: consequences for adult health and human capital. Lancet 371, 340–357. 18206223 10.1016/S0140-6736(07)61692-4 PMC2258311

[mcn12161-bib-0061] Webb‐Girard A. , Cherobon A. , Mbugua S. , Kamau‐Mbuthia E. , Amin A. & Sellen D.W. (2012) Food insecurity is associated with attitudes towards exclusive breastfeeding among women in urban Kenya. Maternal and Child Nutrition 8, 199–214. 20874844 10.1111/j.1740-8709.2010.00272.x PMC6860665

[mcn12161-bib-0062] World Health Organization (WHO) (2002) The Optimal Duration of Exclusive Breastfeeding. Report of an Expert Consultation, WHO: Geneva.

[mcn12161-bib-0063] World Health Organization (WHO) (2003) Global Strategy for Infant and Young Child Feeding, WHO: Geneva.

[mcn12161-bib-0064] World Health Organization (WHO) (2005) Guiding Principles for Feeding Nonbreastfed Children 6 to 24 Months of Age, WHO: Geneva.

[mcn12161-bib-0065] World Health Organization (WHO) (2007) Planning Guide for National Implementation of the Global Strategy for Infant and Young Child Feeding, WHO: Geneva.

[mcn12161-bib-0066] World Health Organization (WHO) (2009) Global Health Risks: Mortality and Burden of Disease Attributable to Selected Major Risks, WHO: Geneva.

[mcn12161-bib-0067] Zulu E.M. , Dodoo F.N. & Chika‐Ezee A. (2002) Sexual risk‐taking in the slums of Nairobi, Kenya, 1993–8. Population Studies 56, 311–323. 12553329 10.1080/00324720215933

